# Emodin protects against intestinal dysfunction and enhances survival in rat model of septic peritonitis through anti‐inflammatory actions

**DOI:** 10.1002/iid3.942

**Published:** 2023-08-28

**Authors:** Zhongjie Hua, Yaqin Wang, Weiping Chen, Wei Li, Jiali Shen

**Affiliations:** ^1^ Department of Emergency Medicine The First People's Hospital of Pinghu Pinghu Zhejiang China

**Keywords:** cecal ligation and puncture, emodin, experimental septic peritonitis, inflammation, intestinal dysfunction

## Abstract

**Background:**

Sepsis is a significant contributor to organ function damage or failure that results in intestinal dysfunction. Emodin (Emo) has received much attention for its notable anti‐inflammatory and antibacterial properties. We aimed to explore the function of Emo on sepsis.

**Methods:**

Sprague Dawley (SD) rats were pretreated with 20 or 40 mg/kg of Emo, followed by using cecal ligation and perforation to establish sepsis models. Hereafter, blood glucose levels, biochemical parameters, and inflammatory cytokines were measured. Additionally, ileal myeloperoxidase (MPO) activity was also measured. Diamine oxidase (DAO) level in plasma, fluorescein isothiocyanate‐dextran 40 (FD‐40) level in serum, bacteria number in blood and peritoneal fluid, histopathological changes of ileum, and tight junction (TJ) protein expressions in ileum were tested to evaluate the barrier function. Furthermore, CD4+ and CD8+ T cells' percentages were evaluated by flow cytometry. Finally, rats' survival rate was calculated as live rats divided by the total number of rats.

**Results:**

Emo pretreatment not only decreased blood glucose level, but also downregulated triglyceride (TG), alanine aminotransferase (ALT), aspartate aminotransferase (AST), serum creatinine (SCr), blood urea nitrogen (BUN) contents for sepsis rats, especially for the high dose of Emo (*p* < .05). Furthermore, Emo inhibited MPO activity and inflammatory factor release (*p* < .05). Crucially, after Emo administration, the barrier function of ileum was enhanced, evidenced by the reduced DAO, FD‐40 levels, decreased bacteria number, alleviated pathological damage in ileum and increased TJ protein expressions (*p* < .05). Rats treated with Emo exhibited increased percentages of CD8+ and CD4+ T cells (*p* < .05), as well as an improved survival rate.

**Conclusion:**

Emo exhibited a remarkable ability to attenuate sepsis by restoring intestinal dysfunction and improving survival rates, and the mechanism was closely related to anti‐inflammatory properties, which provided new solid evidence for the use of Emo in treating sepsis.

## Graphical abstract

This study showed that emodin could attenuate the severe symptoms of sepsis and improve the survival rate of rats. Therefore, emodin may have important application value in the treatment of sepsis.

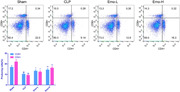

## 1 INTRODUCTION

Sepsis is a syndrome of a series of reactions such as immune dysfunction caused by infection, it can further deteriorate to multiple organ failure, septic shock and even death.^1^ Its mortality for critically ill patients is very high, it has been estimated that sepsis accounted for almost 20% of all deaths worldwide in 2017. One of the typical symptoms of sepsis is intestinal dysfunction. Normally, the intestinal mucosa's barrier function can effectively separate the intestinal cavity from the internal environment.^2^ After sepsis, the intestine is first attacked, resulting in intestinal mucosal damage and enhanced permeability.^3^ Bacteria and toxins will take the opportunity to enter the internal circulation and trigger multiple organ inflammatory reactions. This is consistent with a published study that the intestine is a motor organ leading to multiple organ dysfunction in sepsis.^4^ Therefore, the intestinal tract is the main driving force in the disease progression of sepsis, and enhancing or repairing intestinal barrier may become a goal for sepsis prevention and treatment.^5^ The early treatment methods for sepsis include early diagnosis, antibiotics, controlling the source of infection, fluid resuscitation and supportive treatment,^6^ while these approaches all have some limitations.^7^ Moreover, once the treatment fails, the probability of shock to death will rise sharply.^8^ Therefore, it is an urgent problem to find targeted or adjuvant therapy for sepsis.

Emodin (Emo) is found in various Chinese herbs, has numerous pharmacological functions, including anticancer, antiviral, antioxidant, anti‐inflammatory, antibacterial, immunoregulation, liver protection, and so on.^9^ A published study revealed that Emo can alleviate severe acute pancreatitis in rat models by protecting the intestinal barrier function and preventing the translocation of endotoxin and bacteria.^10^ Published studies reveal that Emo can effectively alleviate jejunum injury^2^ and acute lung injury^11^ in rats that underwent cecal ligation and puncture (CLP). Li et al. revealed that combining Emo and nano silver can exhibit anti‐inflammatory activity to protect against sepsis.^12^ Li et al. discovered that Emo's anti‐inflammatory function can improve mesenteric microcirculation disturbance in rats.^13^ Many studies suggest that Emo has potential application value in treating sepsis or intestinal disease. However, there is little know about the role of Emo on intestinal barrier for septic rats.

Hence, we hypothesized that Emo has an important role in treating sepsis‐caused intestinal dysfunction. By measuring inflammatory factor levels, intestinal barrier function as well as ileal pathological injury markers in sepsis rats, we found that Emo exhibited a protective function on intestinal dysfunction in experimental sepsis, and improve septic animals' survival rate. We will further explore the specific mechanism of Emo in improving intestinal dysfunction caused by sepsis, which may contribute to the further clinical application of Emo in sepsis treatment.

## 2 METHODS

### 2.1 Animals and sepsis modeling

Sprague Dawley (SD) rats (male, 200–230 g) were supplied by Shanghai SIPPR‐BK Laboratory Animal Co., Ltd. (license No. SCXK (Shanghai) 2018‐0006). All animals were raised in SPF conditions, animal room conditions were maintained: relative temperature 20 ± 2°C, relative humidity 60 ± 10%, good ventilation, light and dark alternated every 12 h. The animal project was approved by Animal Experimentation Ethics Committee of Hangzhou Eyong Biotechnological Co., Ltd. Animal Experiment Center(Certificate No. SYXK (Zhe) 2020‐0024). Then, 56 rats were divided into four groups at random (*n* = 14), namely the sham, CLP, Emo‐L as well as Emo‐H groups.

After 1 week of adaptation, CLP‐induced sepsis models were established as the following protocol^14^: all rats were fasted overnight of food, but not water. Then, the rats were anesthetized by isoflurane, and the cecum was carefully pulled out through a median incision in the abdominal wall to avoid damage to the mesenteric vessels. The feces at the upper end of the cecum were squeezed gently to fill the cecal terminals, and the mesenteric surface vessels were separated. After that, a 4‐gauge sterile thread was applied to ligate the blind valve and the cecum midpoint, and puncture the cecal wall with the 21G sterile needle at the middle point of the cecum and the ligation site, resulting in a perforation. The cecum was gently squeezed to ensure smooth perforation, the extruded content was wiped away, then, the cecum was pushed back, and the abdominal cavity was closed. Afterwards, liquid resuscitation was performed using normal saline, and buprenorphine (0.05 mg/kg) was utilized to alleviate pain. The animals from the sham group did not undergo ligation or needle puncture of the cecum, and the other steps were the same as the description above.

Half an hour before CLP operation, the animals from the sham and CLP groups received an intraperitoneal injection of normal saline with an equal volume, meanwhile, the rats in the Emo‐L or Emo‐H groups were given an intraperitoneal injection of 20 or 40 mg/kg of Emo respectively.^2^, ^15^ Emo (B20240) was purchased from Shanghai YuanYe Biotechnology Co., Ltd.

The blood glucose levels of the rats were continuously detected with test paper at 1, 2, 4, 6, 12, and 24 h following CLP operation. Twenty‐four hours after CLP operation, six rats from each group were euthanized, blood samples were obtained via cardiac puncture, and the tissues of intestine and spleen were harvested and stored at −80°C for further use. The remaining eight rats from each group were utilized to monitor survival rate.

### 2.2 Observation of survival rate

The rats' survival rates in the sham, CLP, Emo‐L, and Emo‐H groups were observed and analyzed at 0, 1, 2, 3, and 6 days after operation. Rats' survival rate was calculated as the number of live rats divided by the total number of rats.^16^

### 2.3 Determination of serum biochemical indexes

The blood was centrifuged to obtain the serum. Then, serum alanine aminotransferase (ALT), aspartate aminotransferase (AST), serum creatinine (SCr), blood urea nitrogen (BUN), total cholesterol (TC), triglyceride (TG), high‐density lipoprotein cholesterol (HDL‐C), and low‐density lipoprotein cholesterol (LDL‐C) contents were tested by an Abbott C16000 automatic biochemical analyzer.^17^

### 2.4 ELISA for serum inflammatory cytokines detection

First, the serum samples were taken out. Next, based on the manufacturer's protocols serum inflammatory cytokines (TNF‐α, IL‐1β, and IL‐4) contents were detected by their corresponding ELISA kits (MM‐0132M1, MM‐0040M1, MM‐0191R1).^18^ All the ELISA kits were supplied by Enzyme‐linked Biotechnology.

### 2.5 Detection of myeloperoxidases (MPO) activity in ileum

According to the previous research methods,^19^ the ileal tissue was homogenized using a homogenized buffer after it was weighed. Then, the supernatant of homogenate was obtained through centrifugation. Subsequently, MPO activity in ileum was estimated by an MPO assay kit (MM‐0337R1; Enzyme‐linked Biotechnology).

### 2.6 Count of bacteria in blood and peritoneal fluid

Peritoneal fluid was collected from the rats of each group. According to previous studies,^6^ blood or peritoneal fluid was diluted 10‐fold by sterile phosphate buffered saline (PBS). Then, 20 μL diluted blood or peritoneal fluid was plated on blood agar plates at 37°C for 24 h. Finally, count the number of colony forming units (CFU) in per mL of fluid.

### 2.7 Detection of intestinal barrier function

Inferior vena cava was collected and centrifuged to isolate plasma, and plasma diamine oxidase (DAO) activity in rats was measured using kits purchased from Nanjing Jiancheng Bioengineering Institute (A088‐2‐1) and following the instructions provided.^20^

As per earlier studies,^20^ level of serum fluorescein isothiocyanate‐dextran 40 (FD‐40, 750 mg/kg) was tested to assess intestinal permeability in rats. Specifically, 18 h after CLP surgery, all rats were gavaged with FD‐40 (750 mg/kg). After 6 h, the superior mesenteric vein blood was collected and serum was obtained via centrifugation. The level of serum FD‐40 was detected using a fluorescence spectrophotometer at the wavelength of excitation and emission at 490 and 520 nm.

### 2.8 Histopathological examination of ileum

Ileal tissues were collected and immersed in 4% fixative. Then, the fixed ileum was dehydrated using ethanol, soaked in xylene and embedded in paraffin. Next, the ileum was sequentially sliced, dewaxed, rehydrated and stained with hematoxylin and eosin (HE, H3136, E4009; Sigma) staining for observation under an electron microscope.^21^

### 2.9 Flow cytometry analysis

Splenocytes were isolated from spleen and washed by PBS. Then, the cells were centrifuged to obtain the cell suspension. After counting, the cell concentration was adjusted to 2 × 107/mL. Then, 100 μL of cell suspension was mixed with 2.5 μL of fluorescence‐coupled CD3+, CD4+, and CD8+ antibodies (554833, 550057, 554857), and incubated at 4°C for 0.5 h in the darkness. Afterward, the cells were washed and suspended to view CD4+ and CD8+ T cells by C6 flow cytometer (BD Biosciences) equipped with 488 nm and 640 nm lasers. The obtained data were processed by Kaluza analysis software 2.1 (Beckman Coulter). The fluorescence was measured using FL1 (533/30 bandpass filter), FL2 (585/40 bandpass filter), FL3 (780/60 bandpass filter) and FL4 (675/25 bandpass filter) detectors, respectively. All the fluorescence‐coupled antibodies were supplied by BD Biosciences pharmingen. The gating strategy applied to discern subsets of T cells followed the following steps: first, cells were gated on a forward scatter/side scatter dot plot after gating on CD4 and CD8. Within this gate, CD4+ and CD8+ were calculated absolutely conducted to detect CD4+/CD8+ rate.^22^ In addition, the flow rate for sample acquirement was 35 μL/min, and 10,000 events were counted for every sample.

### 2.10 Immunofluorescence staining

As per published report,^23^ after sectioning, deparaffinization using xylene and rehydration in ethanol, the ileal tissue sections were heated with citrate buffer, treated with 3% H_2_O_2_ and incubated with 3% BSA. After removal of the blocking solution, primary antibodies, including NLRP3 (DF7438), ZO‐1 (AF5145), Claudin‐1 (AF0127), and Occludin (DF7504) were utilized to incubate the sections at 37°C for 3 h. Following washing, the sections were reacted with FITC‐labelled secondary antibodies (1:200, Ab150077; Abcam). Next, DAPI was used to react with the sections in darkness to stain the nuclei. Following mounting, the sections were observed by fluorescence microscope. All the primary antibodies were purchased from Affinity and used at the dilution of 1:200.

### 2.11 Western blot detection

As per published report,^24^ the total protein of ileum was isolated and quantified using BCA kits (pc0020; Solarbio). Next, the protein was resolved by sodium dodecyl sulfate‐polyacrylamide gel electrophoresis (SDS‐PAGE) and transferred onto polyvinylidene fluoride (PVDF) membranes. After that, the membranes were blocked in a blocking solution. Upon incubation with the required primary antibodies (including antibodies anti‐NLRP3 (DF7438), anti‐ZO‐1 (AF5145), anti‐Claudin‐1 (AF0127), anti‐Occludin (DF7504), and anti‐GAPDH (AF7021)), the membranes were cleaned and incubated with second antibodies. Then, the membranes were reacted with ECL and quantified with Image. All the primary antibodies were purchased from Affinity and used at the dilution of 1:1000, except for anti‐GAPDH was diluted at 1:3000.

### 2.12 Statistical analysis

All data was analyzed by SPSS software (Version 22.0, SPSS Inc.), and presented as mean ± SEM. One‐way analysis of variance (ANOVA) and Tukey tests were employed for multigroup comparisons. Kruskal–Wallis H was applied if the variance was not homogeneous. Kaplan–Meier was used to assess the survival rate. *p* < .05 was considered statistically significant.

## 3 RESULTS

### 3.1 Postoperative blood glucose detection

**Figure 1:**
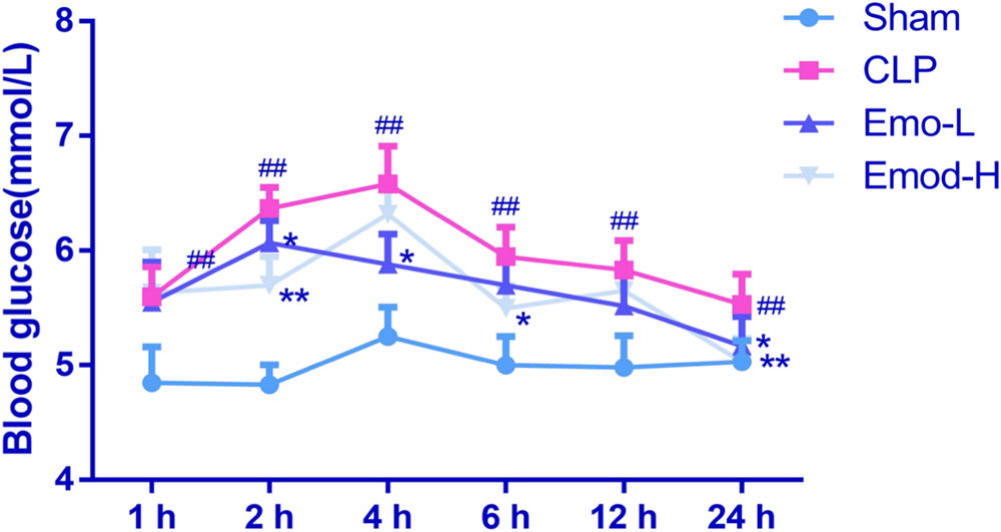
Changes of blood glucose levels at 1, 2, 4, 6, 12, and 24 h after CLP surgery. The number of samples was six. One‐way ANOVA and Tukey tests were utilized for multigroup comparisons. Kruskal–Wallis H was applied when the variance was not homogeneous. ^#^ *p* < .05, ^##^ *p* < .01 versus Sham; *p < .05, **p < .01 versus CLP. CLP, cecal ligation and puncture.

After CLP operation, we began to monitor the changes in blood glucose levels at various time points. As depicted in Figure 1, within 24 h after operation, rats' blood glucose levels in the CLP group initially rose and then gradually decreased, although they remained obviously higher than those of the sham rats. Of note, at both 2 and 24 h, Emo exhibited a dose‐dependent ability to lower blood glucose levels (*p* < .05).

### 3.2 Emo improved serum biochemical parameters in sepsis rats

It could be seen from Table 1 that CLP surgery resulted in elevated TC, TG, HDL‐C as well as LDL‐C levels in rat serum, with a particularly obvious increase observed in TC and TG (*p* < .01). However, treatment with Emo effectively decreased these aforementioned biochemical parameters, with the serum level of TG in the Emo‐H group being notably decreased (*p* < .05).

**Table 1:** Serum TC, TG, HDL‐C and LDL‐C levels ($\bar{\chi }$ ± s).

Group	TC (mmol/L)	TG (mmol/L)	HDL‐C (mmol/L)	LDL‐C (mmol/L)
Sham	1.77 ± 0.12	1.08 ± 0.10	1.20 ± 0.09	0.41 ± 0.04
CLP	2.35 ± 0.14^##^	1.68 ± 0.08^##^	1.13 ± 0.08	0.51 ± 0.04
Emo‐L	2.42 ± 0.20	1.68 ± 0.12	1.23 ± 0.08	0.51 ± 0.05
Emo‐H	2.13 ± 0.21	1.48 ± 0.16*	1.00 ± 0.09	0.54 ± 0.04

*Note*: The number of samples was 6. One‐way ANOVA and Tukey tests were applied for multigroup comparisons.

Abbreviations: CLP, cecal ligation and puncture; HDL‐C, high‐density lipoprotein cholesterol; LDL‐C, low‐density lipoprotein cholesterol; TC, total cholesterol; TG, triglyceride.

^#^ *p* < .05, ^##^ *p* < .01 versus Sham; *p < .05,**p < .01 versus CLP.

Furthermore, the levels of ALT, AST, SCr, and BUN were also assessed. The results exhibited in Table 2 revealed that CLP surgery led to a significant upregulation in serum ALT, AST, SCr, and BUN levels (*p* < .01), which were effectively rescued by Emo administration (*p* < .01).

**Table 2:** Serum levels of ALT, AST, SCr, and BUN ($\bar{\chi }$ ± s).

Group	ALT (U/L)	AST (U/L)	SCr (Umol/L)	BUN (Umol/L)
Sham	12.12 ± 0.85	102.83 ± 8.25	22.51 ± 1.55	6.93 ± 0.81
CLP	40.04 ± 2.31^##^	309.85 ± 21.84^##^	82.64 ± 7.34^##^	15.05 ± 1.36^##^
Emo‐L	33.57 ± 1.45**	219.82 ± 17.94**	63.46 ± 5.18**	12.34 ± 1.01**
Emo‐H	26.07 ± 1.04**	202.92 ± 19.13**	44.18 ± 2.28**	10.17 ± 1.00**

*Note*: The number of samples was 6. One‐way ANOVA and Tukey tests were utilized for multigroup comparisons. Kruskal–Wallis H was applied when the variance was not homogeneous.

Abbreviations: ALT, alanine aminotransferase; AST, aspartate aminotransferase; BUN, blood urea nitrogen; CLP, cecal ligation and puncture; SCr, serum creatinine.

^#^ *p* < .05, ^##^ *p* < .01 versus Sham; *p < .05, **p < .01 versus CLP.

### 3.3 Emo decreased rat ileal MPO activity and serum TNF‐α, IL‐1β, and IL‐4 levels

**Figure 2:**
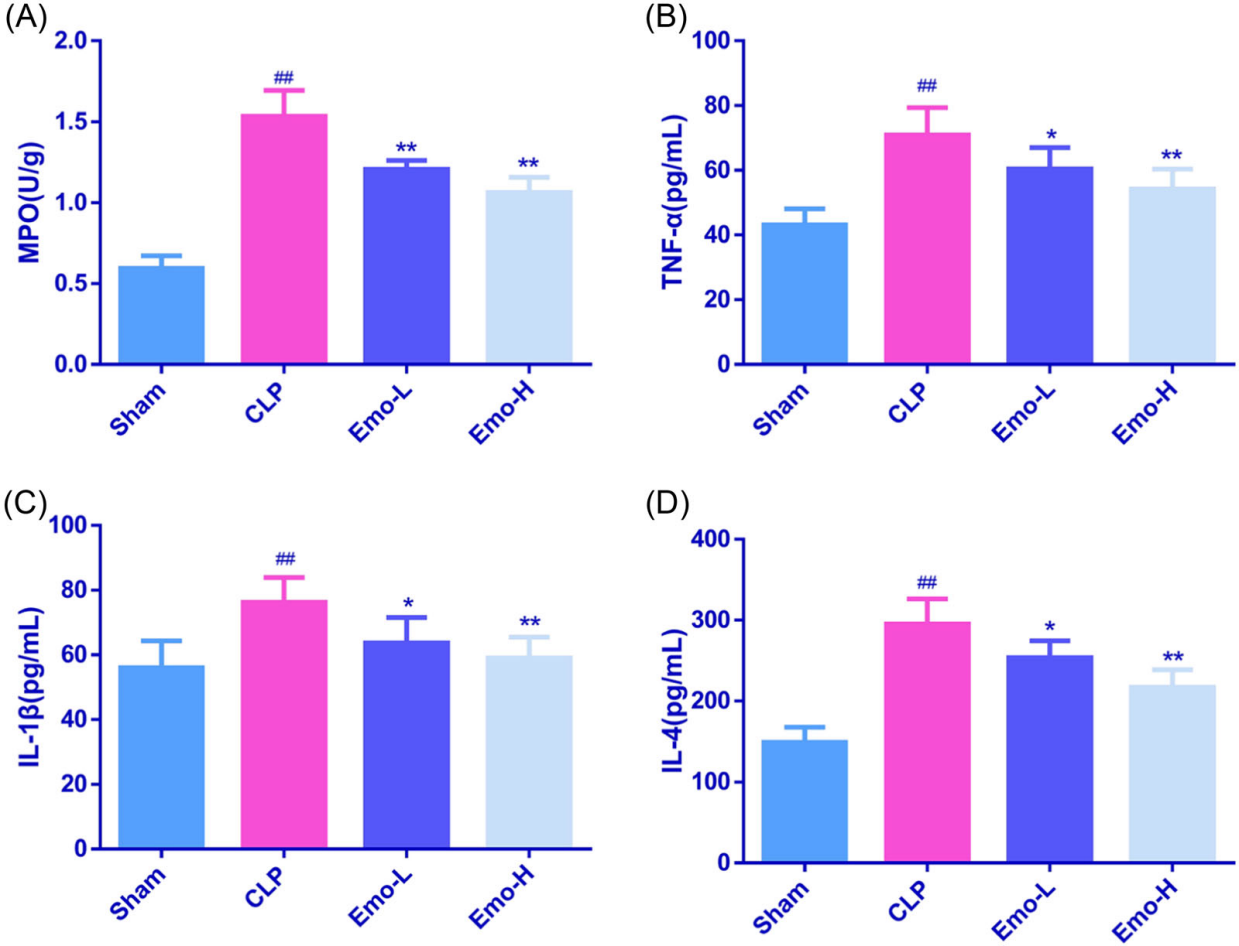
The levels of MPO in ileum (A) and TNF‐α (B), IL‐1β (C), and IL‐4 (D) in serum of rats. The number of samples was 6. One‐way ANOVA and Tukey tests were applied for multigroup comparisons. *p* < .05, ^##^ *p* < .01 versus Sham; *p < .05, **p < .01 versus CLP. CLP, cecal ligation and puncture; MPO, myeloperoxidase.

MPO activity always serves as an indicator of neutrophil infiltration. As depicted in Figure 2A, after CLP, the MPO activity in ileum was markedly elevated (*p* < .01). Nevertheless, after Emo treatment, the MPO activity was decreased observably (*p* < .01). Additionally, we also evaluated postoperative inflammation by detecting the contents of cytokines in rat serum. After CLP modeling, TNF‐α, IL‐1β, and IL‐4 levels were obviously upregulated in rats' serum (*p* < .01). Relative to the CLP rats, the TNF‐α, IL‐1β, and IL‐4 levels in the CLP‐L and CLP‐H groups were decreased notably (Figure 2B–D, *p* < .05).

### 3.4 Emo reduced DAO and FD‐40 levels but improved bacterial translocation

**Figure 3:**
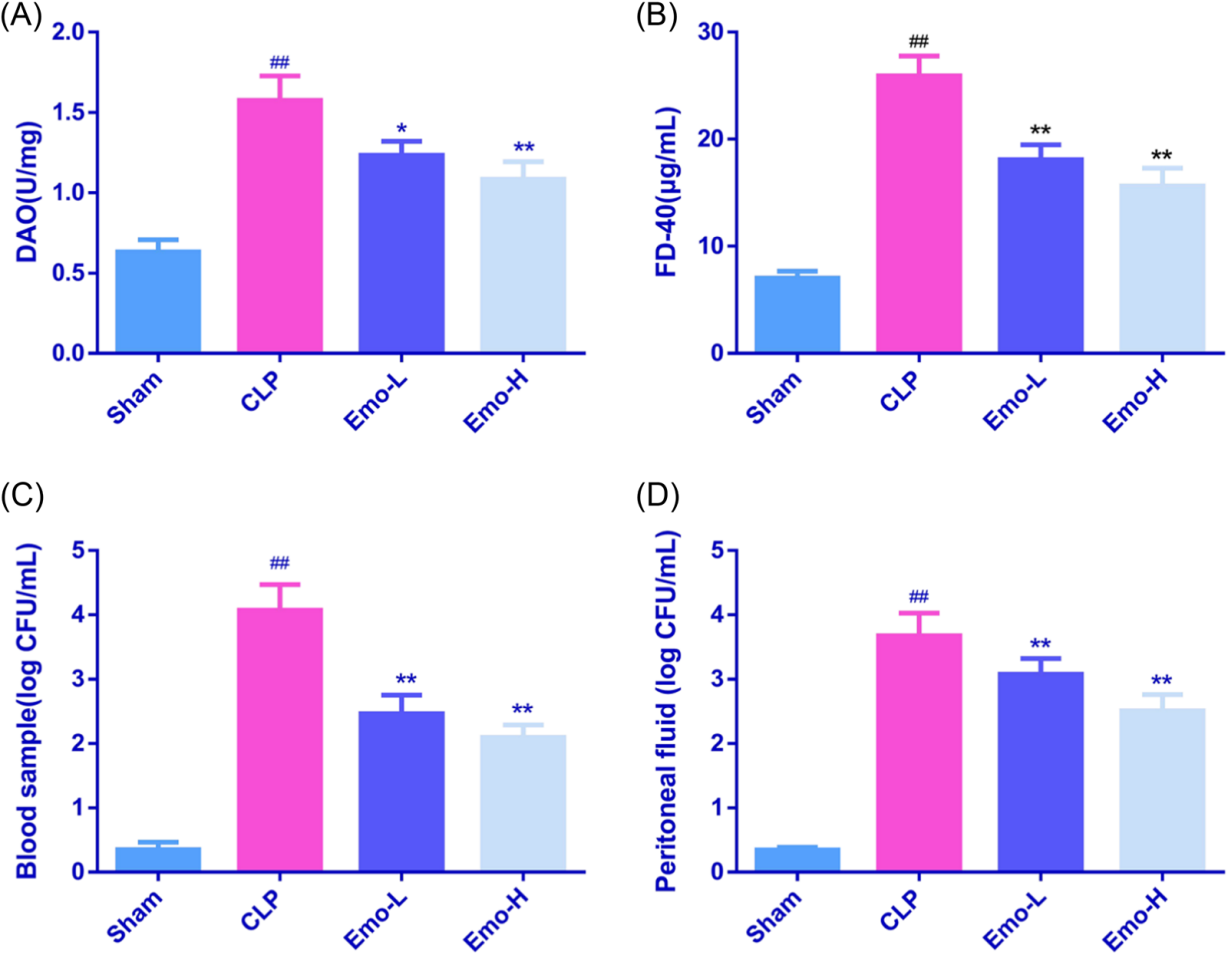
The levels of DAO (A) and FD‐40 (B) in intestinal tract, the number of bacteria in blood and peritoneal fluid of rats (C, D). The number of samples was 6. One‐way ANOVA and Tukey tests were applied for multigroup comparisons. ^#^ *p* < .05, ^##^ *p* < .01 versus Sham; *p < .05, **p < .01 versus CLP. CLP, cecal ligation and puncture; DAO, diamine oxidase.

The results found the CLP rats had significantly higher levels of both DAO and FD‐40 relative to the sham rats (*p* < .01, Figure 3A,B). Nevertheless, after pretreatment with different doses of Emo, both DAO and FD‐40 levels were reduced effectively (*p* < .05). Additionally, bacteria numbers in blood and peritoneal fluid were also counted to further detect bacterial infection in sepsis rats. As shown in Figure 3C,D, CLP treatment resulted in a significant bacterial translocation in rats, with obviously higher numbers of bacteria observed in blood and peritoneal fluid (*p* < .01). However, Emo pretreatment reversed the translocation of bacteria significantly, the numbers of bacteria in Emo‐L and Emo‐H groups were effectively reduced (*p* < .01).

### 3.5 Emo alleviated pathological injury of the ileum in sepsis rats

**Figure 4:**
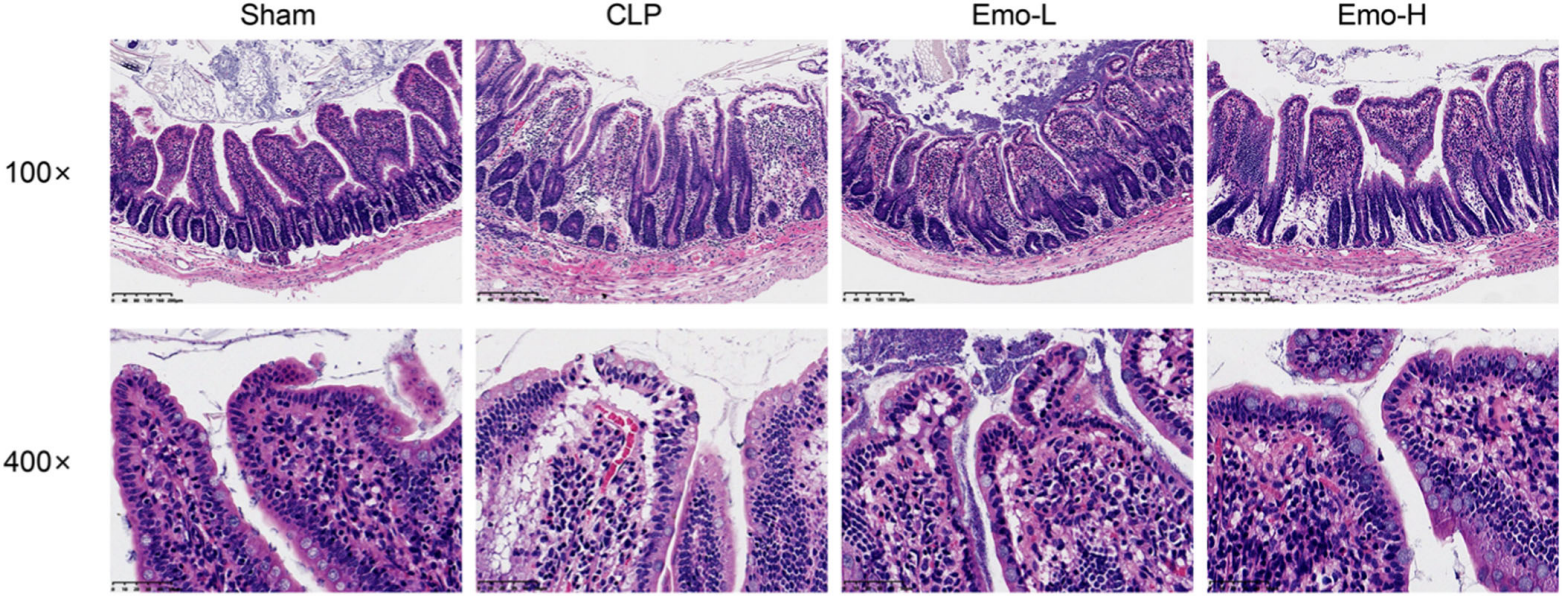
The histopathological changes of ileum of rats were evaluated by HE staining. Scale bar = 200 μm (magnification: ×100), Scale bar = 50 μm (magnification: ×400). The number of samples was 6. HE, hematoxylin and eosin.

From Figure 4, the results of HE staining showed that the sham group has intact intestinal mucosa and arranged orderly intestinal villi. However, in the CLP group, the mucosal tissues of rats were damaged to varying degrees, epithelial degeneration, necrosis, and inflammatory cell infiltration. Interestingly, the pathological damage of the Emo‐L group was alleviated. The epithelial gap of villous apex of intestinal mucosa was narrowed, and the erosion and ulceration were obviously alleviated in the Emo‐H group.

### 3.6 Emo increased CD4+ and CD8+ T cell proportions in rat splenocytes

**Figure 5:**
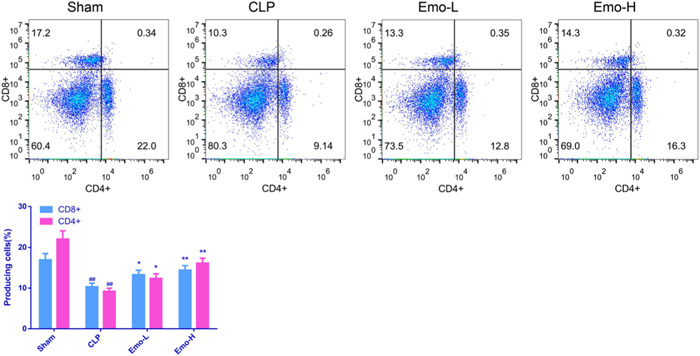
The CD4+ and CD8+ T cell proportions in rat splenocytes. The experiment was replicated three times. One‐way ANOVA and Tukey tests were utilized for multigroup comparisons. ^#^ *p* < .05, ^##^ *p* < .01 versus Sham; *p < .05, **p < .01 versus CLP. CLP, cecal ligation and puncture.

As illustrated in Figure 5, after CLP operation, CD4+ and CD8+ T cell percentages in the splenocytes decreased sharply (*p* < .01). However, in the presence of Emo, the decrease of CD4+ and CD8+ T cell percentage caused by CLP was reversed (*p* < .05). Of note, the effect was more apparent with the higher dose of Emo.

### 3.7 Emo increased tight junction (TJ) protein expression in ileum

**Figure 6:**
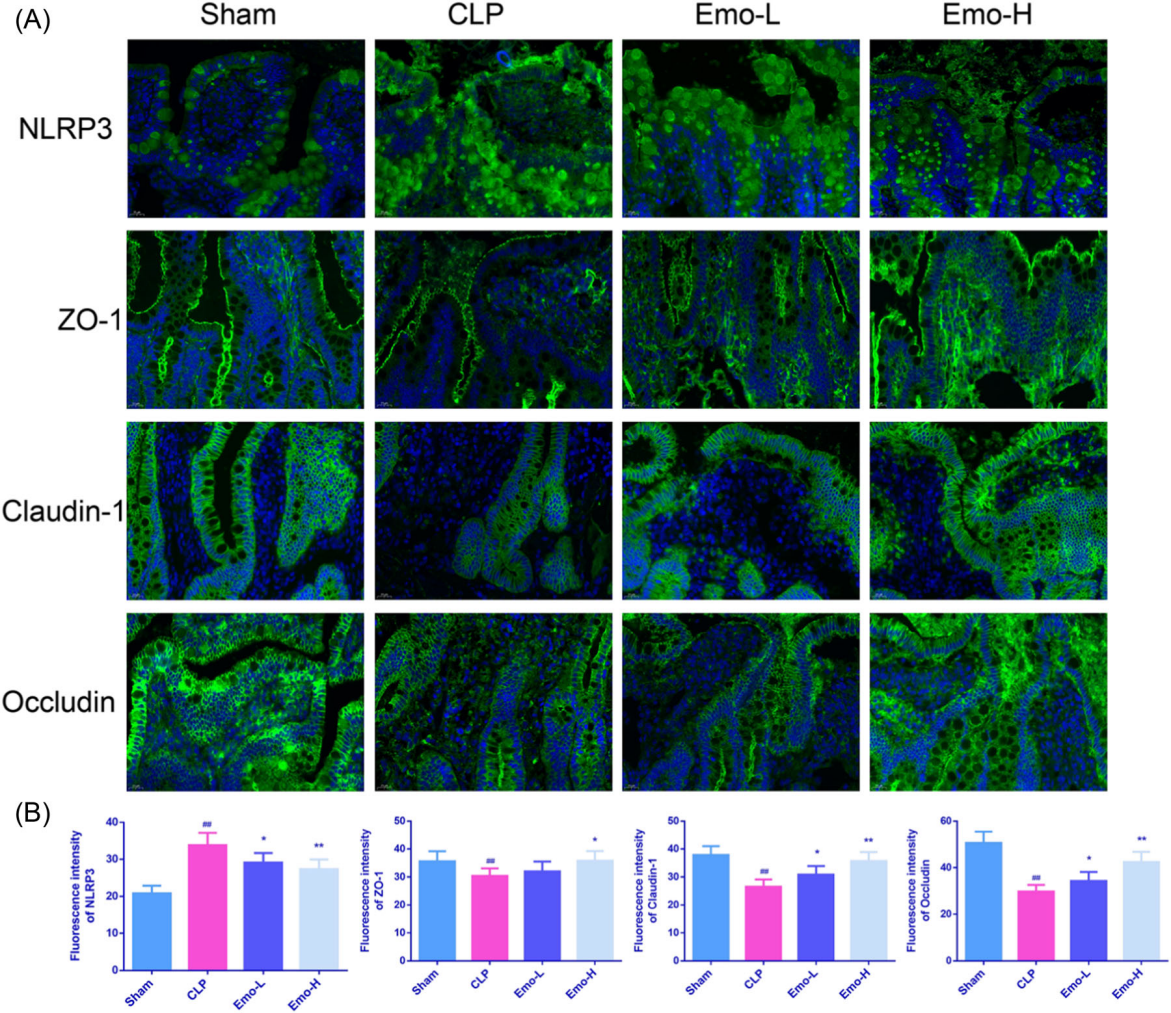
Immunofluorescence staining of NLRP3, ZO‐1, Claudin‐1, and Occludin in ileum (A); semiquantitative analysis of fluorescence intensity (B). Scale bar = 20 μm (magnification: ×400). The number of samples was 6. One‐way ANOVA and Tukey tests were applied for multigroup comparisons. ^#^ *p* < .05, ^##^ *p* < .01 versus Sham; *p < .05, **p < .01 versus CLP. CLP, cecal ligation and puncture.

The immunofluorescence staining results revealed a noticeable increase in NLRP3 fluorescence intensity in the CLP rats, when compared with the sham rats (*p* < .01). Nevertheless, the fluorescence intensity of NLRP3 was decreased obviously in Emo‐L and Emo‐H groups (Figure 6A,B, *p* < .05). On the contrary, relative to the CLP group, ZO‐1 expression was remarkably raised only in the Emo‐H group, while both doses of Emo effectively increased Claudin‐1 and Occludin expressions (*p* < .05).

**Figure 7:**
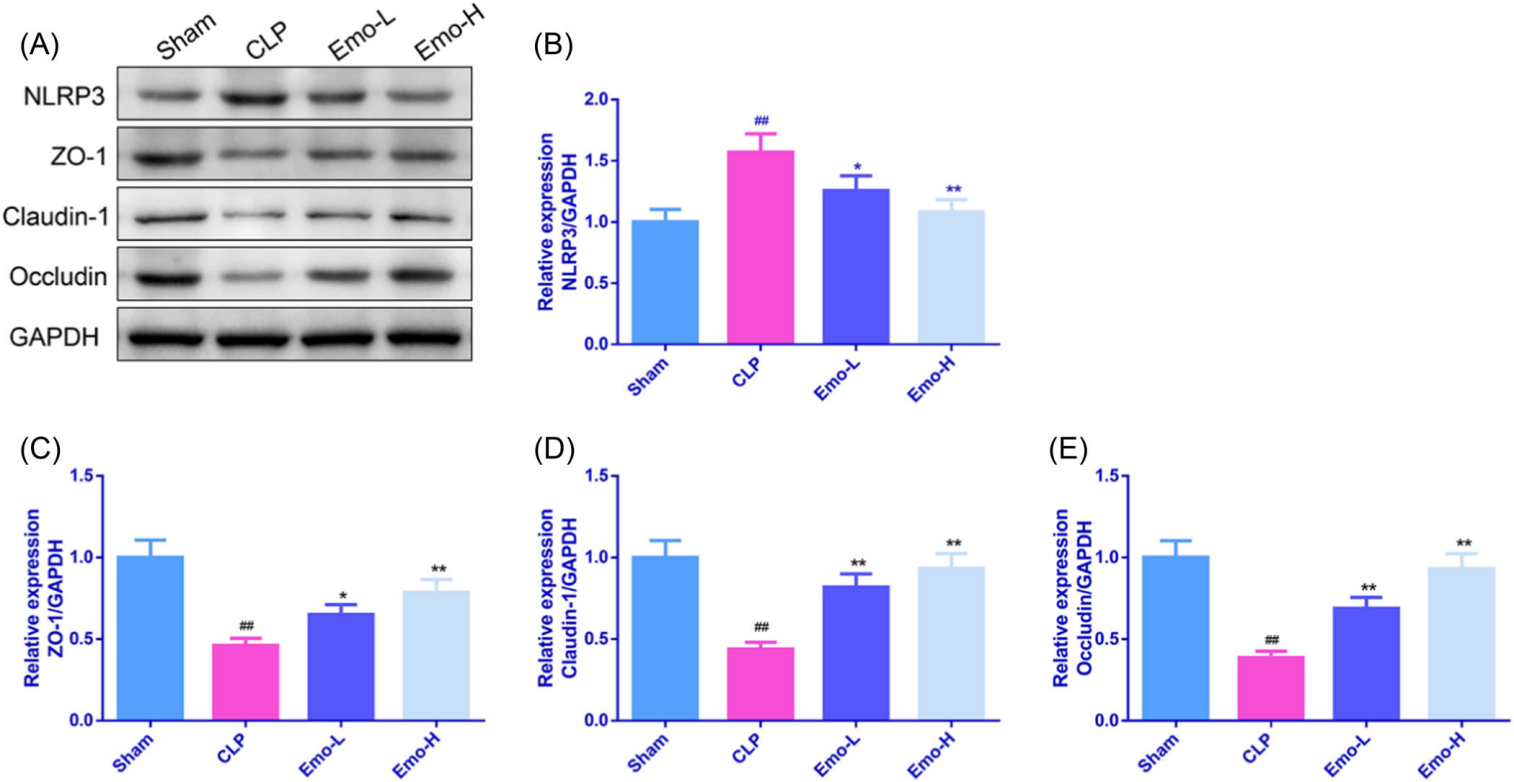
The protein levels and band chart of NLRP3 (B), ZO‐1 (C), Claudin‐1 (D), and Occludin (E) in ileum of rats. The experiment was replicated three times. One‐way ANOVA and Tukey tests were utilized for multigroup comparisons. ^#^ *p* < .05, ^##^ *p* < .01 versus Sham; *p < .05, **p < .01 versus CLP. CLP, cecal ligation and puncture.

The results of Western blot were similar to that of the immunofluorescence staining (Figure 7). The protein level of NLRP3 in the ileum of rats belonging to the CLP group was remarkably elevated, and Emo treatment effectively reverse this result (*p* < .05). Conversely, ZO‐1, Claudin‐1, and Occludin protein levels in the CLP animals were reduced sharply relative to sham rats, while Emo restored the expressions of these proteins (*p* < .05).

### 3.8 Emo improved the survival rate of the sepsis rats

**Figure 8:**
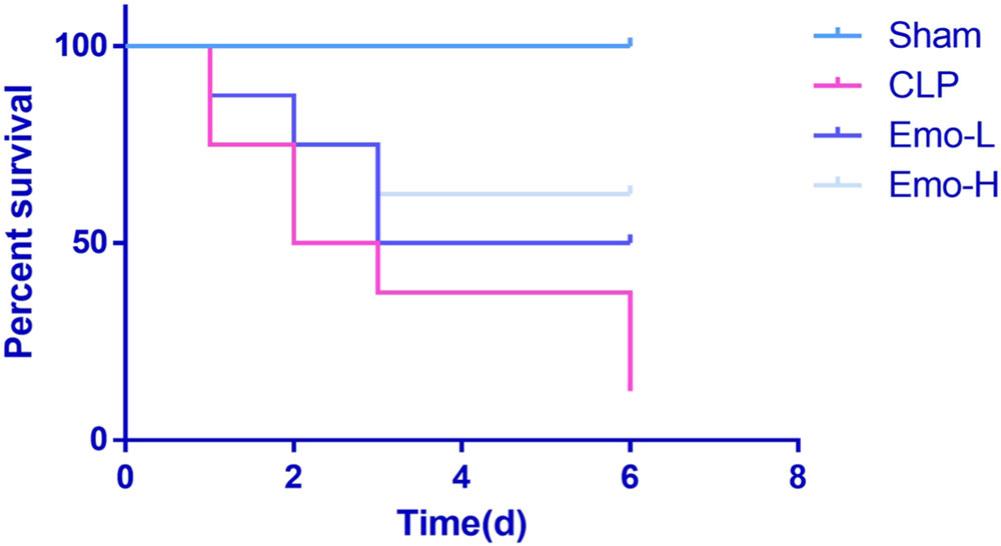
Changes in the rats' survival rate. The number of samples was 8. Kaplan–Meier was sued to assess the survival rate. CLP, cecal ligation and puncture.

As shown in Figure 8, during Days 0–6 after CLP surgery, rats' survival rate was significantly downregulated (*p* < .05), while Emo administration upregulated rats' survival rate.

## 4 DISCUSSION

The annual global growth rate of sepsis is 1.5%, with over 19 million severe cases, and the mortality rate reaching as high as 30%.^25^ Sepsis is an important contributor to high mortality in patients who are ill critically. The pathogenesis of sepsis is complex, including inflammation and immune function disorder.^11^ Studies have shown that sepsis is mainly caused by the inflammatory cascade.^26^ The first organ endangered is the intestine.^3^ Enteric pathogens and endogenous or exogenous toxins that survive after intestinal barrier disruption will invade other organs and circulatory systems through portal vein, intestinal lymph and osmotic reabsorption pathways.^27^ The liver receives venous blood while the lung receives lymph from the gastrointestinal tract, so they may be the further attacked organs of sepsis.^28^ Although there are more in‐depth studies on the disease process and treatment of sepsis and thereby induced intestinal dysfunction, due to the disease characteristics of high mortality, the study of effective treatment of sepsis is still in the bottleneck stage.

It has been reported that in sepsis mice established by CLP, with the changes in corresponding serum indexes, the morphology of lung, liver, kidney, and other organs changed.^29^ After CLP surgery, a variety of endogenous microbial infections produce symptoms similar to human clinical sepsis and peritonitis.^30^ Therefore, CLP operation is the most frequently utilized animal model to study sepsis. In this study, we also performed CLP to induce experimental sepsis for researching the role of intestinal dysfunction on sepsis and evaluating Emo's protective effect against sepsis. The study found that in the CLP group, the blood glucose and serum ALT, AST, TC, TG, SCr, BUN, TNF‐α, IL‐1β, and IL‐4 contents were upregulated; severe pathological damage of intestinal mucosa, intestinal epithelial barrier damage also occurred; CD4+ as well as CD8+ T cell proportion in splenocytes were decreased; moreover, the expressions of major TJ proteins in ileum were decreased. However, Emo can partially reverse the above sepsis injury.

Emo, as a naturally originated active compound, has protective effects against oxidative stress, apoptosis, and inflammation.^31^ It is reported that Emo can modulate Bcl‐2 and Bax protein expression in septic rats, thereby inhibiting intestinal injury.^2^ Zhou et al. have confirmed that Emo can improve intestinal barrier dysfunction in acute pancreatitis by suppressing apoptosis and modulating immune response.^32^ In this study, Emo can significantly reduce serum DAO and FD‐40 levels, which is beneficial to protect the integrity of intestinal epithelial barrier. In addition, immune dysfunction is the predominant feature of patients with sepsis.^29^ In studying experimental sepsis, it was found that T cell proportions were also affected, such as CD4+ as well as CD8+ T cells, which were major components of the immune dysfunction of sepsis.^33^ Studies have demonstrated that both rhubarb and Emo could enhance immune function by upregulating the secretion of CD4+ lymphocytes and restoring the CD4+/CD8+ ratio.^34^ Our study found that the proportions of CD4+ as well as CD8+ T cells induced by sepsis decreased significantly, while Emo treatment greatly reduced this reduction effect. This may contribute to its reported immunoregulation effect.

Some researchers reported that anti‐inflammatory genes targeting intestinal dysfunction can improve intestinal dysfunction and improve sepsis survival rate.^35^ From this research, we suggested that Emo displayed a protective effect against sepsis mainly via its anti‐inflammatory effect, which helped to inhibit the inflammatory response in septic animals and improve intestinal barrier integrity by regulating TJ‐related protein expressions. TJ protein, as its name suggests, is a key protein that tightly connects epithelial cells and can prevent macromolecules from passing through epithelial cells.^36^ Therefore, intestinal barrier's protective function depends on TJ barrier's integrity. Zhang et al. showed that TJ protein expressions, including ZO‐1, claudin‐1, and occludin, decreased significantly in intestinal inflammatory diseases.^37^ This study found that ZO‐1, Claudin‐1, and Occludin protein expressions were downregulated in septic ileum, but Emo reversed this situation. Li et al. believed that in the pathogenesis of sepsis, the organism will produce anti‐inflammatory and proinflammatory reactions, making the organism in an immune disorder.^6^ Many studies confirmed Emo can decline TNF‐α expression.^38^ The results of this paper were consistent with the above research, and Emo downregulated inflammatory factors contents. We speculated that in sepsis, Emo may not only downregulate pro‐inflammatory factors' levels, but also upregulate anti‐inflammatory factors' levels, so as to prevent or treat sepsis through its anti‐inflammatory effect.

However, a limitation of the study is the lack of cellular experiments. In the future, we will use lipopolysaccharide‐induced intestinal epithelial cells to further verified the key findings of the study.

## 5 CONCLUSION

Our research demonstrated that rat sepsis model induced by CLP showed intestinal dysfunction, produced a series of inflammatory reactions, and decreased survival rate. Emo can partially reverse the severe symptoms of sepsis, to attenuate the sepsis‐induced intestinal dysfunction. In addition, the study revealed that Emo may enhance intestinal mucosal barrier function through its anti‐inflammatory effect. Therefore, Emo, as a component of traditional Chinese medicine, may have important application value in treating sepsis.

## AUTHOR CONTRIBUTIONS

**Zhongjie Hua**: Conceptualization (lead); writing—original draft (lead); formal analysis (lead); writing—review and editing (equal). **Yaqin Wang**: Software (lead); writing—review and editing (equal). **Weiping Chen**: Methodology (lead); writing—review and editing (equal). **Wei Li**: Writing—original draft (supporting); writing—review and editing (equal). **Jiali Shen**: Conceptualization (supporting); writing—original draft (supporting); writing—review and editing (equal). All authors have read and approved the final manuscript.

## CONFLICT OF INTEREST STATEMENT

All authors declare no conflict of interest.

## ETHICS STATEMENT

The animal project is approved by the Animal Experimentation Ethics Committee of Hangzhou Eyong Biotechnological Co., Ltd. Animal Experiment Center (Certificate No. SYXK (Zhe) 2020‐0024).

## Data Availability

The data that support the findings of this study are available from the corresponding author upon reasonable request.
